# Spatial transcriptomic characterization of COVID-19 pneumonitis identifies immune circuits related to tissue injury

**DOI:** 10.1172/jci.insight.157837

**Published:** 2023-01-24

**Authors:** Amy R. Cross, Carlos E. de Andrea, María Villalba-Esparza, Manuel F. Landecho, Lucia Cerundolo, Praveen Weeratunga, Rachel E. Etherington, Laura Denney, Graham Ogg, Ling-Pei Ho, Ian S.D. Roberts, Joanna Hester, Paul Klenerman, Ignacio Melero, Stephen N. Sansom, Fadi Issa

**Affiliations:** 1 Nuffield Department of Surgical Sciences, University of Oxford, Oxford, United Kingdom.; 2 Department of Pathology,; 3 Department of Internal Medicine, and; 4 Department of Immunology and Immunotherapy, Clínica de la Universidad de Navarra, Pamplona, Spain.; 5 Medical Research Council Human Immunology Unit, Radcliffe Department of Medicine, Medical Research Council Weatherall Institute of Molecular Medicine, University of Oxford, Oxford, United Kingdom.; 6 Department of Cellular Pathology, Oxford University Hospitals NHS Foundation Trust, Oxford, United Kingdom.; 7 Nuffield Department of Medicine, University of Oxford, Oxford, United Kingdom.; 8 CIBERONC, Madrid, Spain.; 9 Center for Applied Medical Research, Pamplona, Spain.; 10 Kennedy Institute of Rheumatology, University of Oxford, Oxford, United Kingdom.

**Keywords:** COVID-19, Inflammation, Cellular immune response, Chemokines, Molecular pathology

## Abstract

Severe lung damage resulting from COVID-19 involves complex interactions between diverse populations of immune and stromal cells. In this study, we used a spatial transcriptomics approach to delineate the cells, pathways, and genes present across the spectrum of histopathological damage in COVID-19–affected lung tissue. We applied correlation network–based approaches to deconvolve gene expression data from 46 areas of interest covering more than 62,000 cells within well-preserved lung samples from 3 patients. Despite substantial interpatient heterogeneity, we discovered evidence for a common immune-cell signaling circuit in areas of severe tissue that involves crosstalk between cytotoxic lymphocytes and pro-inflammatory macrophages. Expression of *IFNG* by cytotoxic lymphocytes was associated with induction of chemokines, including *CXCL9*, *CXCL10*, and *CXCL11*, which are known to promote the recruitment of CXCR3^+^ immune cells. The TNF superfamily members *BAFF* (*TNFSF13B*) and *TRAIL* (*TNFSF10*) were consistently upregulated in the areas with severe tissue damage. We used published spatial and single-cell SARS-CoV-2 data sets to validate our findings in the lung tissue from additional cohorts of patients with COVID-19. The resulting model of severe COVID-19 immune-mediated tissue pathology may inform future therapeutic strategies.

## Graphical abstract

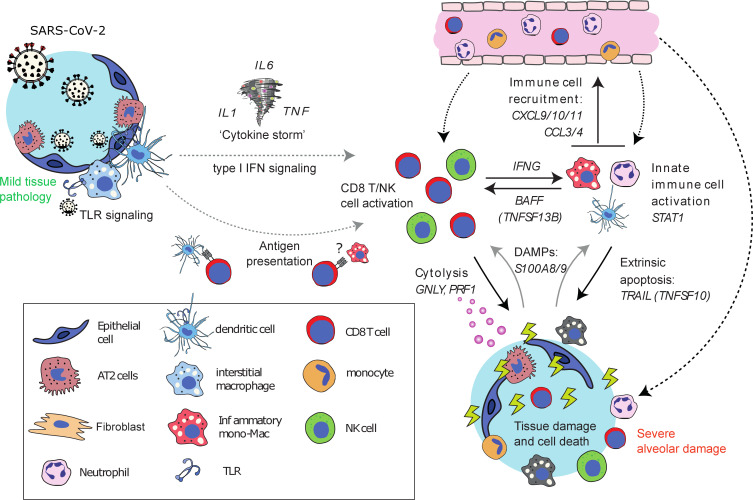

## Introduction

Death after severe SARS-CoV-2 infection is largely related to the antiviral response and immune-mediated lung injury (1). Histopathologically, COVID-19 pneumonitis is associated with diffuse alveolar damage (DAD), fibrosis, leukocytic infiltrates, and microvascular thromboses (2–4). Features of DAD include alveolar wall thickening, interstitial expansion, hyaline membrane deposition, and pneumocyte hyperplasia. Researchers have begun to describe the transcriptomic profiles of lung pathology, although these have been largely designed to assess the cellular impact of SARS-CoV-2 infection (5–7). To our knowledge, later-stage severe organ pathology is not consistently associated with high levels of infection or active viral replication (8, 9). In lung tissue from severe cases, the variability in the detection of SARS-CoV-2 RNA or antigen supports a model of inflammation-perpetuated disease (5, 9). The immune contributors and biological pathways associated with the widespread severe alveolar injury remain unclear; therefore, a greater understanding of the pathological features of COVID-19 would complement the growing knowledge of both tissue- and blood-based immune profiles (10).

Advanced spatial profiling techniques provide the tools to identify the distribution of proteins and RNAs in situ, allowing the dissection of biological processes (BPs) in and around specific histological features of interest (11, 12). We used an advanced, multiplexed, ISH tissue-analysis platform to generate detailed transcriptomic profiles of multiple spatially discrete areas in lung samples from 3 patients with COVID-19, with a focus on the spectrum of DAD severity. Uniquely, these tissues were obtained via open sampling at the point of death, which ensured high-quality RNA analyses and avoided the caveats associated with late autopsies. Application of network-based approaches allowed for deconvolution and visualization of the cell types and immune-cell signaling phenotypes present in the patients’ tissues. After integration of our results with those from other spatial and single-cell sequencing studies, we propose a cellular model for the active immune processes in the lung during severe COVID-19.

## Results

### Immune cell infiltration is associated with severe local tissue damage in COVID-19.

**Figure 1:**
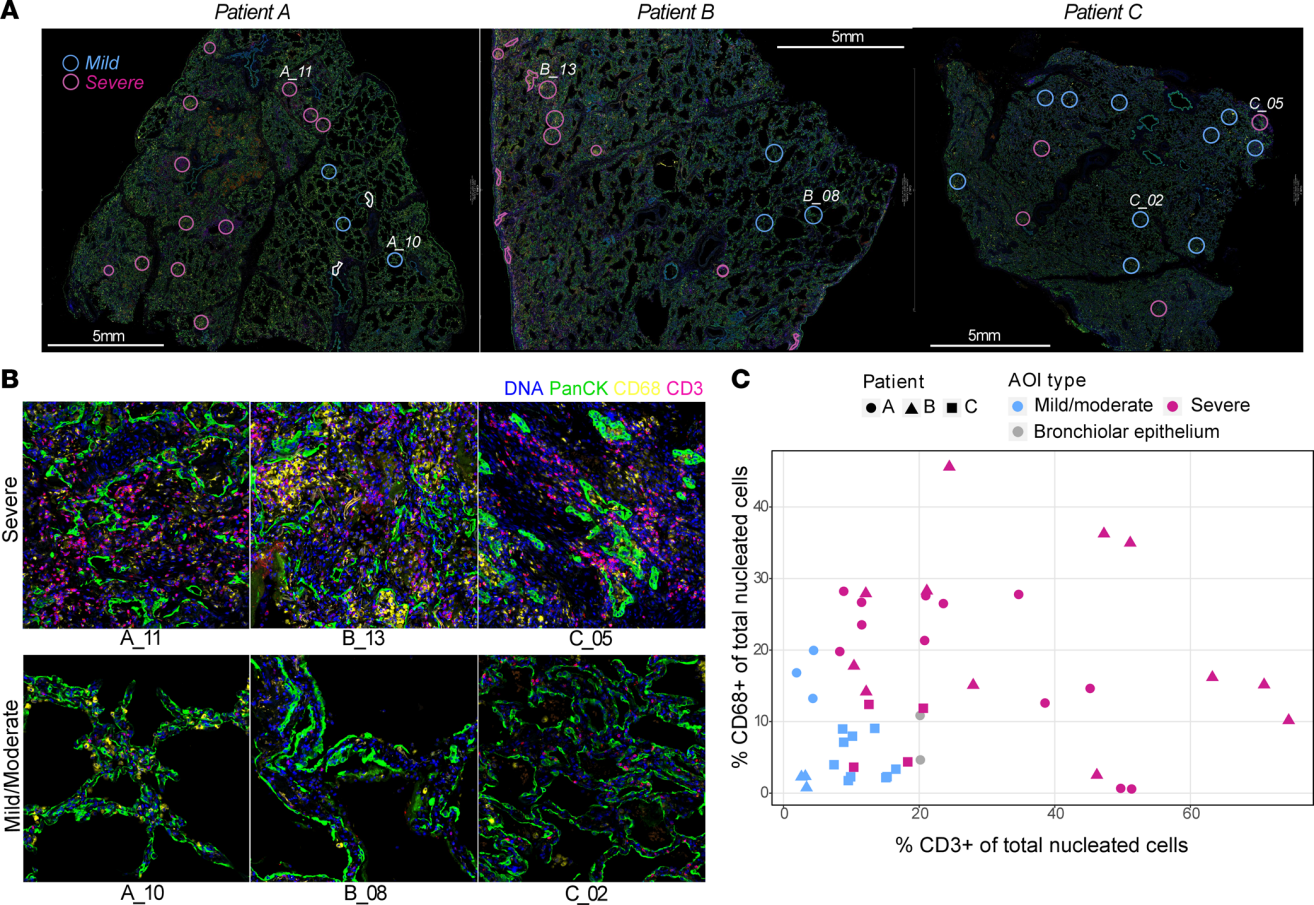
A spectrum of DAD and inflammation was observed within and across COVID-19 lung biopsy specimens. Selection and annotation of areas of DAD in COVID-19 lung tissues for transcriptomic analysis. (**A**) Merged immunofluorescence (IF) images of the lung samples from patients A, B, and C (scale bars: 5 mm; panCK, green; DNA, blue; CD3, red; CD68, yellow). AOIs (*n* = 47) selected for transcript profiling are highlighted (mild to moderate, blue circles; severe pathology, magenta circles), of which 46 passed quality control after sequencing. Labeled areas correspond to higher magnification examples in **B**. (**B**) Representative IF images of AOIs demonstrating the morphology and immune infiltrate observed within areas of severe and mild to moderate DAD. AOIs spanned, on average, 0.2 mm^2^ (range, 0.05–0.33 mm^2^) with exclusion of empty space. (**C**) The proportions of CD3^+^ and CD68^+^ cells of total nucleated cells were derived from the immunofluorescence imaging, plotted for each AOI and colored by histological severity (mild to moderate, blue; severe, magenta) or inclusion of bronchiolar epithelium (grey). AOIs are annotated by patient: patient A, circle; patient B, triangle, patient C, square.

The histological and immune-cell landscape within COVID-19 lung tissue from 3 patients with fatal disease was investigated to establish the extent of intratissue variation in cellular pathology (mean specimen area, 1.78 cm^2^; Supplemental Table 1; supplemental material available online with this article; https://doi.org/10.1172/jci.insight.157837DS1). Each sample featured a spectrum of DAD, from mild to severe, comprising pneumocyte hyperplasia, hyaline membrane formation, and interstitial expansion. There was a nonuniform distribution of immune infiltrates (Figure 1, A and B, Supplemental Figure 1, and Supplemental Table 2). Histological features beyond DAD included vacuolated macrophages, edema, vascular thrombi, and squamous metaplasia. Bulk detection for SARS-CoV-2 nucleocapsid (N) RNA by qPCR in patients’ lung samples showed low viral loads (Supplemental Table 2), and there was significant N protein within hyaline membranes and pneumocytes in patient B (Supplemental Figure 2, B and D). By contrast, in patients A and C, only small areas of weak viral N protein expression were detected in the alveolar space and ciliated bronchiolar epithelium (Supplemental Figure 2, A, C, and D). Immunofluorescent staining for CD3, CD68, and pan-cytokeratin (panCK) enabled the identification of lymphocytes, macrophages, epithelial cells, and general tissue architecture (Figure 1B). On the basis of this staining, we selected 46 areas of interest (AOIs) with a broad range of inflammatory features (Figure 1, B and C). DAD histopathological severity was assessed by 2 independent pathologists, and each AOI was categorized as having either mild to moderate injury with some conservation of alveolar architecture or severe injury with a loss of alveolar structure and substantial inflammation. In addition, for each AOI, we quantified the number of CD3^+^ lymphocytes, CD68^+^ macrophages, and cells (by nuclear stain). The SARS-CoV-2 N protein positivity of adjacent tissue from sequential sections was also recorded. AOIs with severe DAD were associated with higher numbers of T cells, although CD68^+^ macrophage numbers were not consistently increased (Figure 1C and Supplemental Figure 2, G and H); however, histopathological severity was not consistently associated with higher levels of RNA for the SARS-CoV-2 N protein (Supplemental Figure 2, E and F).

**Figure 2:**
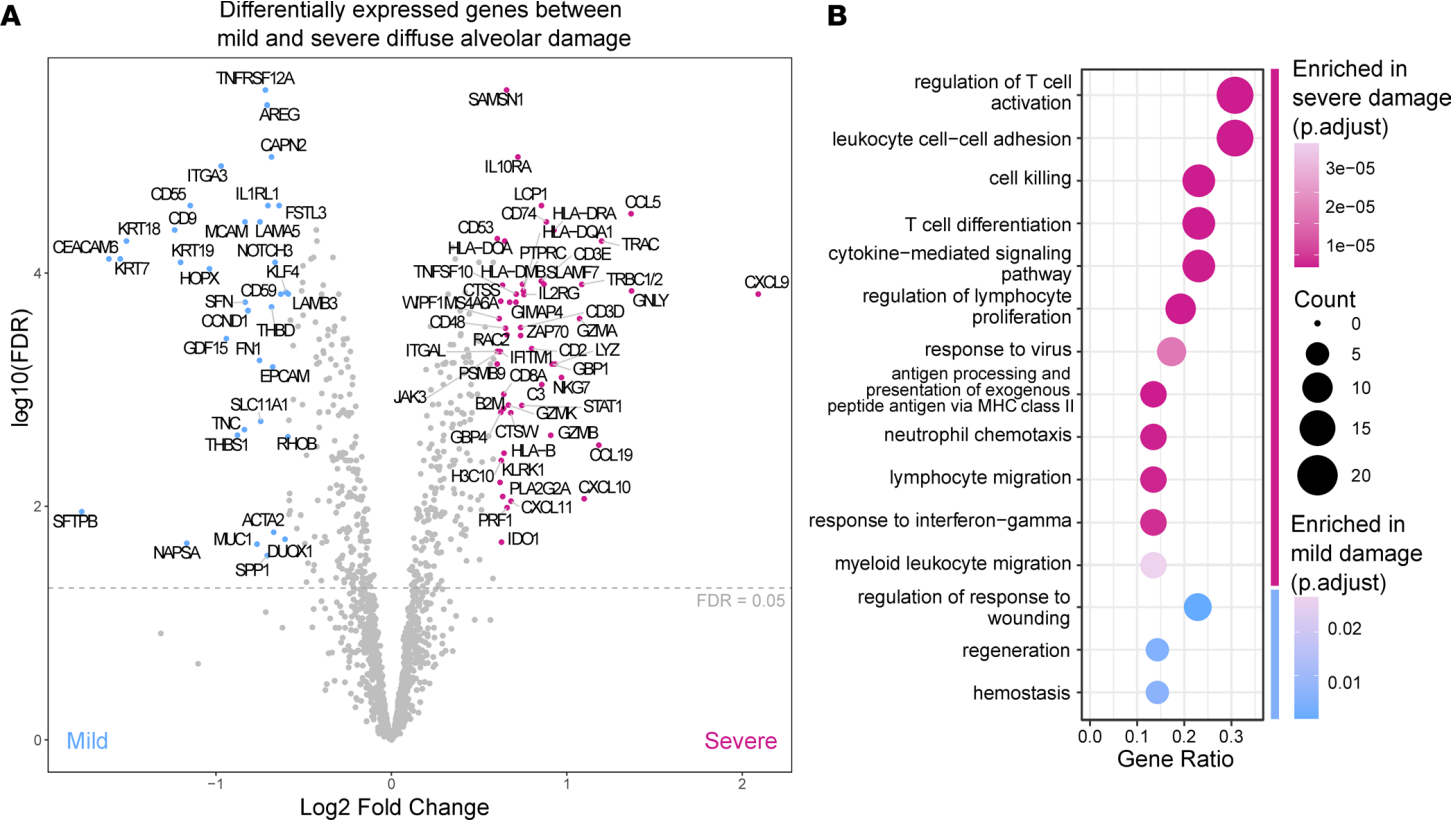
Severe alveolar damage was associated with the upregulation of immune transcripts. (**A**) Differential gene expression between areas of severe and mild to moderate damage. Colored and annotated genes have a fold-change expression greater than 1.5 and a BH adjusted *P* < 0.05 calculated by testing with linear mixed models for repeated measures to compare severity while accounting for repeated sampling of each tissue (mild vs. severe, *n* = 16 and 28, respectively). (**B**) Selected GO BPs significantly overrepresented in genes differentially expressed between mild and severe areas of damage (BH corrected *P* < 0.05, 1-sided Fisher’s exact test). Also see Supplemental Table 3 for differentially expressed genes and all overrepresented pathways.

To profile the pathological pathways active in each AOI, we applied a multiplexed ISH approach to quantitate the expression of a curated panel of more than 1800 genes enriched in immune targets and augmented with a COVID-19–specific gene set. Use of robust quantile normalization resulted in comparable distributions of gene expression and levels of housekeeping genes across the heterogenous AOIs (Supplemental Figure 3). Differential expression analysis of mild and severe DAD across all sampled AOIs (*n* = 16 mild; *n* = 28 severe) identified 56 genes with significantly higher expression in severe pathology (Benjamini–Hochberg [BH] adjusted *P* < 0.05; |fold change| >1.5), including those encoding chemokines (namely, *CXCL9*, *CXCL10*, *CXCL11*, *CCL19*, and *CCL5*), cytotoxic molecules (namely, *GZMA*, *GZMB*, *GZMK*, *PRF1*, *GNLY*, *LYZ*, *NKG7*, and *KLRK1*), complement factor (*C1QB*), and proteins involved in antigen processing and presentation (namely, *CD74, HLA* genes, and *CTSS*) (Figure 2A). Genes upregulated in the severe areas showed a significant overrepresentation (BH adjusted *P* < 0.05) of gene ontology (GO) BP terms related to T cell activation and differentiation, antigen presentation, cytokine production, cytotoxicity, and response to IFN-γ (Figure 2B). By contrast, genes enriched in areas of mild damage (*n* = 40; BH adjusted *P* < 0.05; |fold change| >1.5) had a significant overrepresentation (BH adjusted *P* < 0.05) of pathways associated with wound healing, regeneration, and hemostasis (Figure 2B).

### Network analysis implicates CD8^+^ T cells, mononuclear phagocytes, and active TLR, IFN, and IL-1 signaling in COVID-19 lung inflammation.

**Figure 3:**
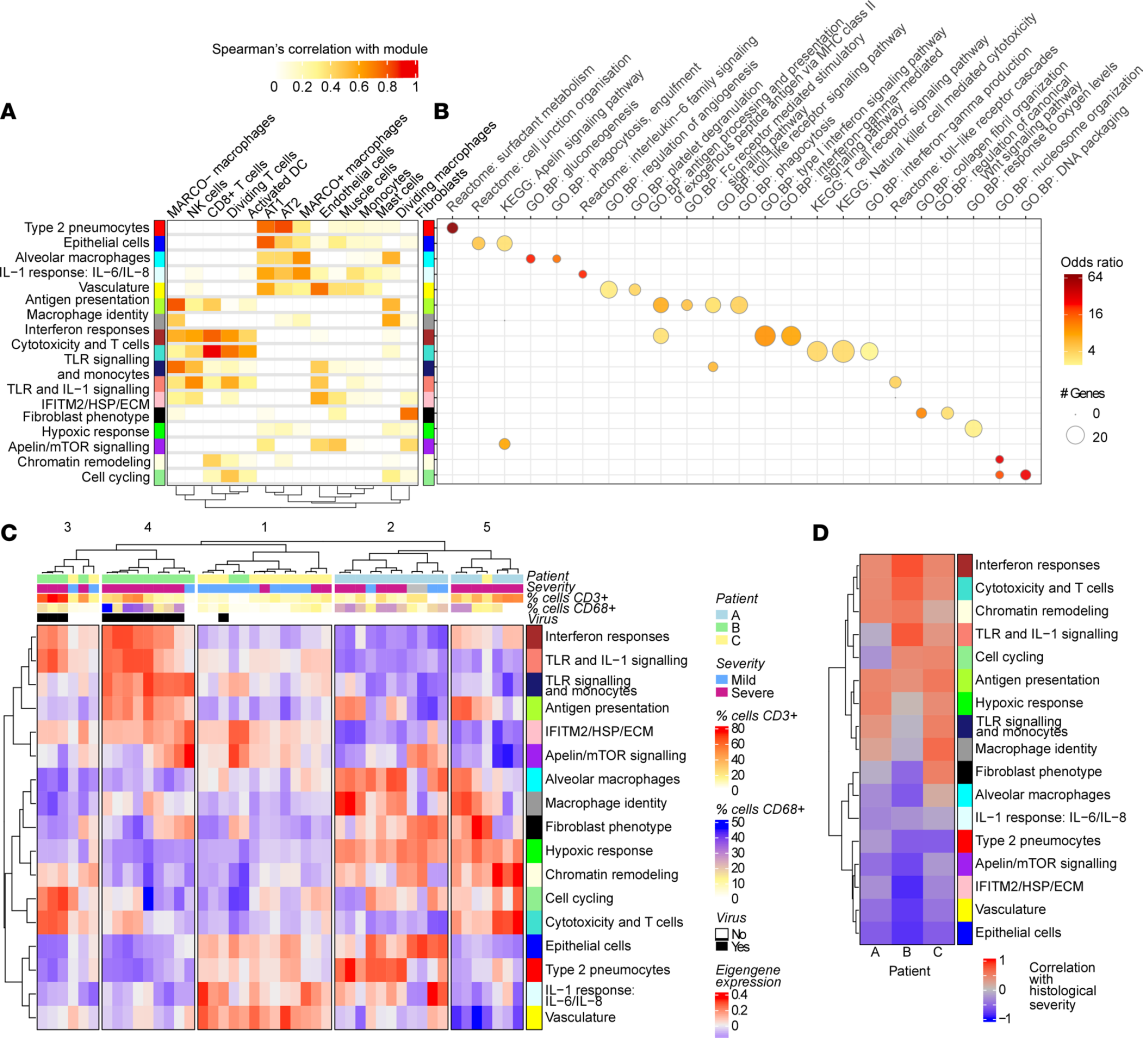
Identification and characterization of gene modules with spatially heterogenous expression in COVID-19 lung tissue. Application of WGCNA to spatial transcriptomic data (*n* = 46) identified 17 modules of coexpressed genes (see also Supplemental Figure 3). (**A**) Correlation between estimated cell-type abundance (as determined by cell deconvolution; see Supplemental Table 6) and WGCNA module eigengene expression (all AOIs; positive Spearman’s correlation values are shown). (**B**) Selected GO BP, KEGG, and reactome pathways significantly overrepresented in the detected modules (BH adjusted *P* < 0.05; 1-side Fisher’s exact tests; see also Supplemental Table 5). (**C**) WGCNA module eigengene expression is shown for each AOI (see also Supplemental Table 4). Sampled AOIs are annotated with patient identity, the severity of damage, adjacent virus antigen presence, and the percentage of CD3^+^ and CD68^+^ cells of total nucleated cells. No severity grade was given to 2 AOIs sampling bronchiolar epithelium. Hierarchical clustering of the 46 AOIs by expression of the WGCNA module eigengenes identified 5 spatial groups with distinct patterns of module expression. (**D**) The severity of tissue damage was correlated to module eigengene expression separately for the AOIs from each patient (Pearson’s correlation). vRNA, viral RNA.

To perform an unbiased exploration of the cellular and phenotypic variations present in the set of the profiled AOIs, we used weighted gene correlation network analysis (WGCNA) (13). This analysis identified 17 distinct modules of co-expressed genes (*n* = 27–266 genes per module; median, *n* = 88) (Supplemental Figure 4, A–D, and Supplemental Table 4). We began a systematic characterization of the identified modules by investigating their association with specific cell types. To do so, we correlated expression of the module eigengenes (the representative module expression patterns) with separate cell-type abundance estimates that were determined for each AOI by automatic cell-type deconvolution analyses (14) (Figure 3A and Supplemental Figure 5, A–D). To confirm the involvement of specific cell types, we examined the correspondence of the cell-type abundance predictions with the expression of a curated set of known cell-type marker genes (Supplemental Figure 6, A and B). We generally saw a good agreement between the marker genes and the automatic predictions of cell-type abundance. However, cell-deconvolution methods did not detect the presence of neutrophils, despite known changes in circulating phenotype or numbers.

On closer inspection of neutrophil-associated genes, the expression of *MPO*, *ELANE*, and *CTSG* correlated with each other, suggesting that neutrophils may be present in some of the AOIs (Supplemental Figure 5A and Supplemental Figure 6, A and B). To help resolve this discrepancy, we applied mass cytometry imaging to sequential sections and examined the areas aligned with selected severe AOIs. This analysis revealed the presence of a substantial number of CD15^+^ neutrophils in an area of severe damage from patient B, whose tissues also showed strong staining for the SARS-CoV-2 N protein. Additionally, this analysis confirmed the expected presence of CD68^+^ macrophages and CD8^+^ T cells within severe areas, with only a small number of CD4^+^ cells being detected (Supplemental Figure 7). Next, we identified BPs and pathways overrepresented among the gene members of each WGCNA module (Figure 3B and Supplemental Table 5). On the basis of these analyses, we named each module according to its cell-type associations and biological pathway enrichments.

As expected for lung tissue, we found a set of stromal modules representing (a) epithelial cells (containing *EPCAM*)*,* (b) type 2 pneumocytes (containing the surfactant-encoding genes *SFTPB*, *SFTPC*, and *SFTPD*), (c) fibroblasts (“fibroblast phenotype,” containing *COL1A1*, *COL3A2*, *COL5A1*, and *THY1*), and (d) vasculature (containing *CDH5*, *THBD*, and *ENG*), which all showed corresponding pathway and cell-type associations (Figure 3, A and B, and Supplemental Figure 4B). We found 3 modules that showed a high correlation with the presence of CD68^+^ cells (Figure 3A and Supplemental Figure 4, B and C). These comprised (a) an alveolar macrophage module that also displayed high expression correlation with the macrophage receptor *MARCO* and enrichment for the “phagocytosis, engulfment” GO BP; (b) a macrophage identity module that also showed high expression correlation with the mannose receptor *MRC1* (a marker of alternative M2 macrophages) and *SIRPA*; and (c) an antigen presentation module that showed a high correlation with predicted inflammatory monocyte–derived macrophage (monoMac) cell abundance (Figure 3B and Supplemental Figure 5, C and D) along with high expression correlation with *SIGLEC1*, *C1QA*, and *C1QB* (Supplemental Figure 4B). We also noted an *IFITM2*/HSP/ECM module that contained *MERTK* and *PDGFRA,* and a module with pathway enrichments for Apelin/mTOR signaling.

Critical COVID-19 is associated with massive lung immune-cell infiltration, and in keeping with this, we discovered modules of genes with clear associations with lymphocytes and mononuclear phagocytes. The cytotoxicity and T cells module was associated with the GO IFN-γ production BP, the IFN responses module (Supplemental Figure 4C), as well as the Kyoto Encyclopedia of Genes and Genomes (KEGG) T cell receptor signaling and NK-mediated cytotoxicity pathways (Figure 3B). The expression of this module was positively associated with predicted presence of CD8^+^ cytotoxic T cells, NK cells, and activated DCs (Figure 3A), as well as the presence of CD3^+^ cells (Supplemental Figure 4C). This module contained known T cell markers, including *CD3D*, *CD3E, CD2*, and *CD8A*, as well as genes associated with cytotoxicity, such as *PRF1* and *GNLY* (Supplemental Figure 4B). The antigen presentation module was associated with areas of high CD68 expression, as indicated by immunofluorescence microscopy (Supplemental Figure 4C), and showed positive associations with the macrophage identity, cytotoxicity and T cells, IFN response, and TLR signaling–related gene modules (Supplemental Figure 4D). The TLR signaling and monocytes module contained the gene encoding the classical monocyte marker *CD14* along with *CD163* (Supplemental Figure 4B).

In addition, we discovered several modules that contained genes associated with innate inflammation, including an IFN responses module, a TLR and IL-1 signaling module, and an “IL-1 response: IL-6/IL-8” module, which were named according to their pathway enrichments (Figure 3B). The vasculature and IL-1 response: IL-6/IL-8 modules contained genes associated with mature neutrophils (namely, *ELANE*, *MME*, *MPO*, and *CTSG*) (Supplemental Figure 4B). The vasculature module was also associated with the GO BP platelet degranulation pathway (Figure 3B). Finally, we found 3 modules that were associated with more general cellular processes. The presence of these modules, which we termed cell cycling, chromatin remodeling, and hypoxic response, was indicative of immune cell proliferation and oxygen stress.

### Severe alveolar damage in COVID-19 is linked with myeloid cell antigen presentation, T cell cytotoxicity, and expression of the CXCL9/10/11 IFN response genes

We next sought to better understand the variation in cellular and immune phenotypes that was present across the tissues and sampled AOIs. To do so, we hierarchically clustered the AOIs by expression of the WGCNA module eigengenes. This analysis revealed 5 groups of AOIs with different transcriptional signatures and associations with severity, patient, and cell composition (Figure 3C and Supplemental Figure 8). This clustering broadly split patients A and B into 2 distinct groups with differences in the cytotoxicity and T cells module expression, whereas sample AOIs of patient C had a consistent transcriptomic profile. Spatial groups were numbered from 1 to 5 according to their association with severe damage: spatial group 1 contained the lowest proportion of severe AOIs, and spatial group 5 consisted of only severe alveolar damage.

We performed a cellular phenotype network analysis to investigate the correlations between WGCNA eigengene expression (transcriptional phenotype), chemokine and cytokine expression (immune signaling), and the predicted cell-type abundances (cell-type identity) in each of the 5 spatial groups (Supplemental Figure 9). This analysis provided evidence that severe tissue damage in these patients with COVID-19 involved an ensemble of interacting and proliferating immune cells in which myeloid cells such as inflammatory monoMac and DCs were activated by TLR-mediated signaling, expressed IL-1 and IFN-α, and presented antigen to cytotoxic lymphocytes driving the production of IFN-γ and a specific cassette of chemokines and cytokines. This cassette included high expression of CXCL9, CXCL10, and CXCL11, factors known to act via CXCR3 to promote immune cell chemotaxis, extravasation, and activation (15), as well as IL-32, which stimulates TNF-α and IL-6 secretion from macrophages (16), and CCL19, which acts via CCR7 to promote DC and central memory T cell migration (17).

#### Cytotoxic lymphocytes, IFN-γ signaling, myeloid cell activation, and TRAIL are associated with severe DAD and are reproducible features of critical COVID-19.

Next, we sought to assess the extent to which the discovered features of severe DAD might represent common features of critical COVID-19. Within AOIs from each of the 3 patients, severe DAD was consistently associated with the expression of the IFN responses, cytotoxicity and T cells, chromatin remodeling, antigen presentation, and TLR signaling (TLR and IL-1 signaling or TLR signaling and monocytes) module eigengenes (Figure 3D and Supplemental Figure 4E). The IL-1 response: IL-6/IL-8 module did not show an obvious correlation with DAD severity, whereas greater expression of gene modules associated with epithelial cells and vasculature was associated with the lowest DAD scores (Figure 3D and Supplemental Figure 4E).

**Figure 4:**
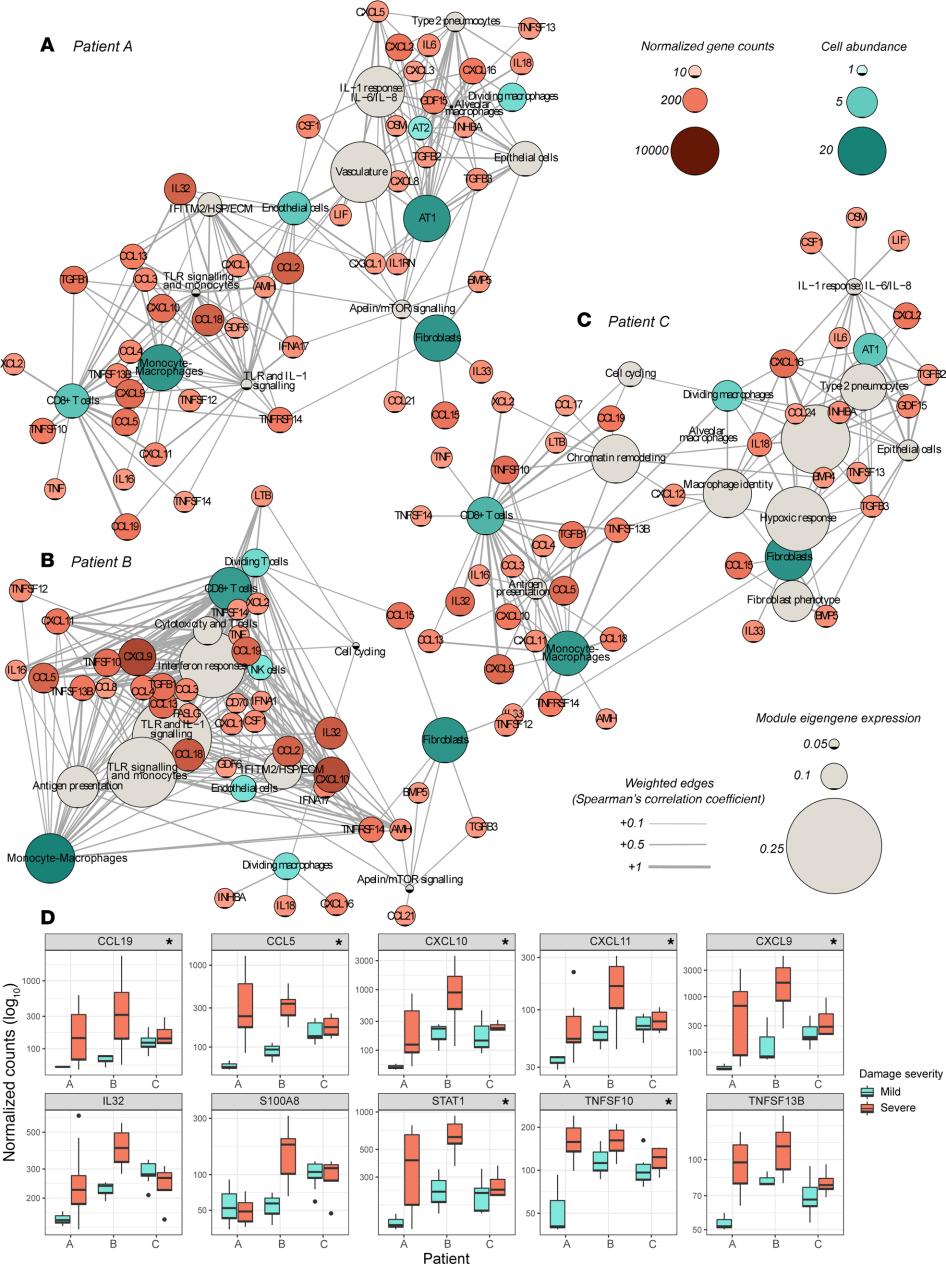
Within-patient analysis of spatially associated cellular phenotypes. (**A**–**C**) The cellular phenotype network analysis diagrams show the correlations (Spearman’s *P* < 0.05) among the WGCNA module eigengene expression values, predicted cell-type abundances, and secreted cytokine expression for the 3 patients (see *Methods* for node inclusion criteria). (**D**) The expression of selected genes in each of the 3 patients for mild (blue) and severe (red) areas of alveolar damage (median, IQR, and outliers are >1.5 times the IQR from the hinge). Asterisks indicate BH adjusted *P* < 0.05 and fold-change >1.5 in differential expression analysis with linear mixed models for repeated measures between mild and severe areas of damage (mild vs. severe, *n* = 16 and 28, respectively).

To investigate commonalities and differences of the tissue pathology of the 3 patients in more detail, we repeated the cellular phenotype network analysis separately for the AOIs of each patient. This analysis demonstrated that a core association among monoMacs; CD8^+^ T cells; the expression of IFN-γ target chemokines *CXCL9*, *CXCL10*, and *CXCL11*; the expression of *IL32*; the expression of the regulator of extrinsic apoptosis *TRAIL* (*TNFSF10*); and the expression of B cell–activating factor *BAFF* (*TNFS13B*) was present in the lung tissues of all 3 patients (Figure 4, A–C).

To help prioritize immune signaling factors involved in severe DAD, we ranked secreted immune signaling factors (and *STAT1*) according to their expression level and upregulation in the severe areas. In addition to the aforementioned signaling molecules, the analysis also highlighted the possible involvement of *STAT1*, *CCL5*, *CCL18*, *CCL13*, *CCL4*, *CCL3*, *CCL21*, *TGFB1*, *CXCL12*, and *CXCL2* (Supplemental Figure 10).

To refine our understanding of the cellular sources of involved cytokines, we directly correlated their expression with that of specific cell-type markers. *CXCL9, CXCL10*, and *CXCL11* showed the strongest correlation with CD8^+^ T cell marker genes, whereas *STAT1, TRAIL* (*TNFSF10*), and *BAFF* (*TNFSF13B*) also showed a strong association with *CD74* and *CD16A* (*FCGR3A*), which are associated with nonclassical monocytes and have been used as markers of inflammatory monoMacs in COVID-19 (7) (Supplemental Figures 10 and 11). Cell deconvolution could not identify B or plasma cell signatures, but *BAFF* correlated with *CD19* expression across the tissue samples from the 3 patients, suggesting B cell involvement in sites of injury (Supplemental Figure 10).

The elevated expression of *TRAIL* in regions of severe DAD is consistent with a previous reports of apoptosis-pathway upregulation in alveolar areas in late-stage COVID-19 (6). The correlation of antigen presentation–related genes with DAD severity (Figure 3D, Supplemental Figure 4E, and Supplemental Figure 9) suggested a directed cytotoxic response, but we did not find an obvious association between the severity and the levels of SARS-CoV-2 N protein or RNA (Supplemental Figure 2, E and F). This suggested that CD8 T/NK cell activation might be triggered by antigen-presenting cell presentation or pattern recognition receptor (e.g., TLR) recognition of viral antigens from abortive infection, of self-antigens, of damage-associated molecular patterns (DAMPs), or a combination thereof. In support of the possible involvement of DAMPs, we noted that endogenous DAMP-encoding genes such as *CCL5* showed robust (if not necessarily upregulated) expression in areas with severe DAD.

Finally, we inspected the expression of key genes of interest with the mild and severe AOIs of each patient. This analysis confirmed a consistent and significant elevation of expression of *CCL19*, *CCL5*, *CXCL9*, *CXCL10*, *CXCL11*, *STAT1*, *TRAIL* (*TNFSF10*), and *BAFF* (*TNFSF13B*) in the areas of severe DAD in the 3 patients examined (Figure 4D). Expression of *IL32* was elevated in the severe AOIs of 2 of the patients, whereas *S100A8* only showed increased severity-associated expression in patient B, in whom its expression was likely neutrophil derived (Supplemental Figure 10).

**Figure 5:**
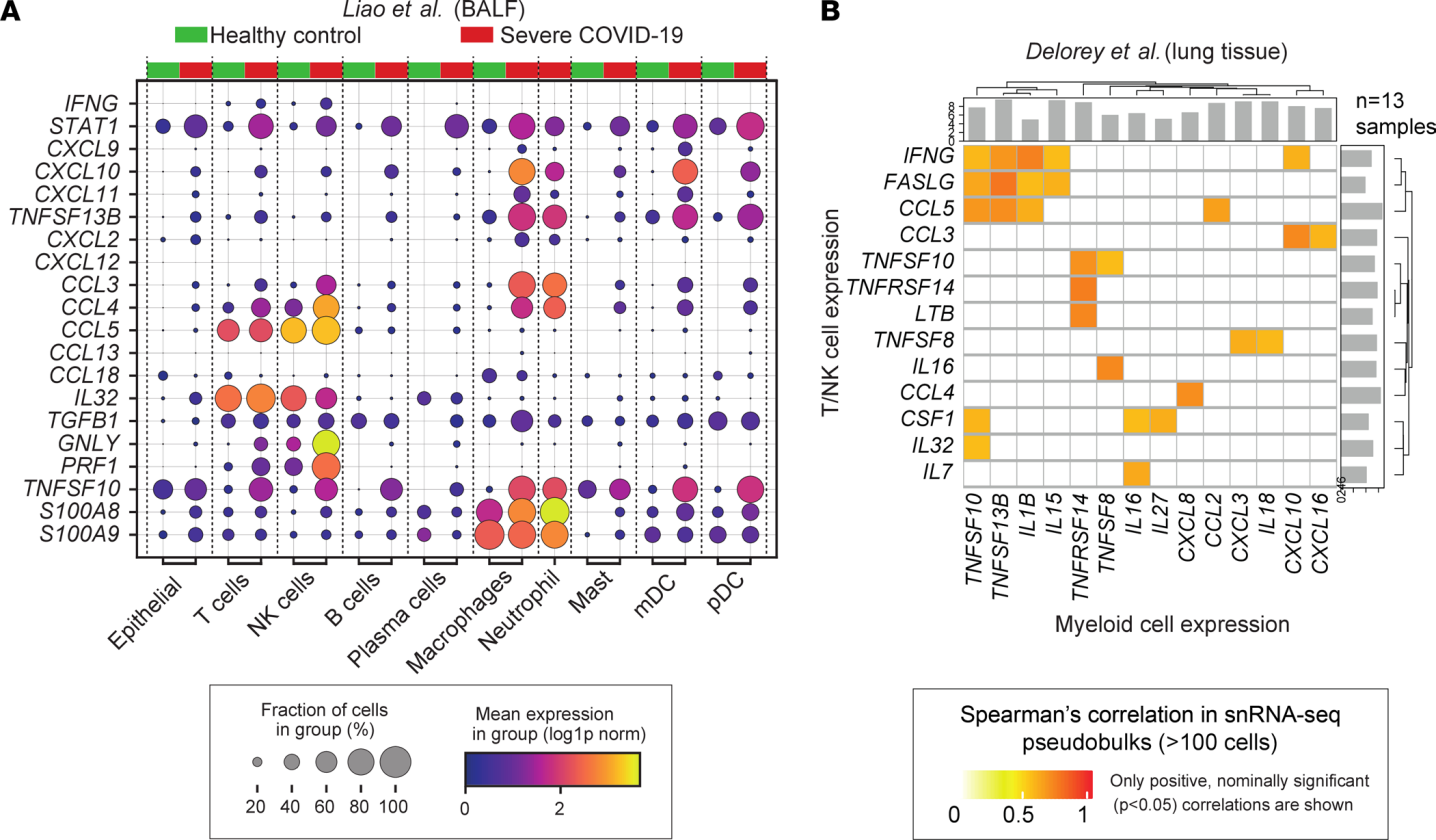
IFN-γ production by cytotoxic lymphocytes is associated with severe tissue damage in COVID-19. (**A**) Analysis of the expression of genes of interest in a published BALF-sample, single-cell data set comprising 4 healthy donors and 6 patients with severe COVID-19 (18). (**B**) Spearman’s correlations between cytokine expression in T and NK and myeloid nuclei pseudobulks constructed from a published single-nuclei atlas of the lung tissue of SARS-CoV-2–infected autopsy donors with COVID-19 (*n* = 13) (7). mDC, myeloid dendritic cell; pDC, plasmacytoid dendritic cell; snRNA-seq, single-nucleus RNA sequencing.

To determine if the IFN-γ signaling, discovered *CXCL9/10/11*–containing cytokine cassette, cytotoxic lymphocytes, *TRAIL*, *BAFF*, and endogenous DAMP expression are replicable features of lung pathology in critical COVID-19, we investigated the expression of the relevant genes in available community data sets. Examination of single-cell data from bronchoalveolar lavage fluid (BALF) samples (18) confirmed an increase in *IFNG* expression in T and NK cells in patients with COVID-19 relative to healthy control tissue, along with increased expression of *CXCL9*, *CXCL10*, *CXCL11*, and *BAFF* in myeloid cells and neutrophils (Figure 5A). We also noted a broad increase in *STAT1* and *TRAIL* expression, along with induction of *CCL3* and *CCL4* expression in macrophages and neutrophils. The cytotoxic molecule-encoding genes *GNLY* and *PRF1* were upregulated in NK and T cells and neutrophils, which were not captured in the healthy control tissue, constituted an additional source of *S100A8/9*. The cellular sources and expression levels of these genes were consistent with those observed in other lung tissue single-nuclei and BALF single-cell data sets (Supplemental Figure 12, A and B). Inspection of a previously published spatial data set of alveolar tissue (6) (which did not discriminate between mild and severe DAD) confirmed upregulation of *STAT1*, *CXCL10*, *BAFF*, and *TRAIL* in COVID-19 (Supplemental Figure 12C). Increased expression of these genes in COVID-19 was also observed, albeit weakly, in a bulk analysis of macrophages purified from BALF (Supplemental Figure 12D).

Together, these previously published observations and our targeted spatial analysis suggest the existence of a cellular circuit in which IFN-γ production by T and NK cells drives CXCL9/10/11 and BAFF production by myeloid cells in COVID-19. If such a circuit exists, we reasoned that the expression levels of these cytokines should co-vary in the relevant cell types in lung tissue across individuals with COVID-19. To investigate this possibility, we analyzed single-nuclei data from the lung tissue of 16 SARS-CoV-2–infected autopsy donors who had COVID-19 (7) (Supplemental Figure 13). In keeping with the existence of the proposed circuit, levels of *IFNG* in per-donor T and NK nuclei pseudobulks were significantly correlated (*P* < 0.05) with the levels of *TRAIL*, *BAFF*, and *CXCL10* in the paired myeloid nuclei pseudobulks (Figure 5B).

## Discussion

Within the lung, COVID-19 manifests in a wide spectrum of DAD and fibroproliferation. The histopathological features are nonspecific, and there are no clear findings that differentiate SARS-CoV-2 from a number of other respiratory viral infections, particularly those developing after infection with other betacoronaviridae such as SARS-CoV and MERS-CoV (19–24). Although much has been learned from analysis of blood and BALF (10, 18, 25–29), study of tissue is needed to understand the cellular causes of COVID-19–associated lung damage. Further insight has been gained from single-cell and single-nuclei approaches (7, 30), but these approaches do not retain spatial information that is vital for deciphering the interplay between different cell types. Initial applications of spatial proteomics and transcriptomics have started to reveal the spatial landscape of lung damage in COVID-19 (6, 7), but molecular details of the signaling circuits that perpetuate pathology remain to be fully elucidated.

In this study, we sought to generate new insights by applying network-based analysis approaches to the analysis of rich spatial transcriptomics data generated with the Nanostring GeoMx Digital Spatial Profiler (DSP) platform. To do so, we used correlation networks to integrate WGCNA module eigengenes, cytokine gene expression levels, and computationally predicted cell-type abundances. Use of this flexible and extensible cellular phenotype network analysis approach uncovered new links among the cell types, biological pathways, and cytokines that are associated with lung tissue damage in severe COVID-19.

We found substantial differences in the cellular and molecular pathologies of the lung tissue sampled from the 3 patients we studied. Tissue from patient A displayed a stronger signature of type 2 pneumocyte and alveolar macrophages. This patient also had the highest expression of a gene module associated with hypoxic response, an observation that was not unexpected given the detection of low oxygen response and p53 stress pathways in the BALF of patients critically ill with COVID-19 (31). In contrast with tissue from the other 2 patients, the tissue from patient B showed the strongest IFN response and TLR and IL-1 signaling signatures, which corresponded with immunohistochemical evidence of high levels of viral infection and the presence of neutrophils. These findings are is consistent with the idea that neutrophil extracellular trap formation (or NETosis) may contribute to ongoing inflammation in some patients with COVID-19 (32). Finally, the samples from patient C showed a lower expression of IFN and hypoxic response signatures and were distinguished by elevated expression of a vasculature-associated gene module. Despite these broad differences, our initial within-patient analysis revealed a shared association of severe DAD in COVID-19 with IFN signaling, cytotoxicity and T cells, cell proliferation, and antigen presentation–related genes.

**Figure 6:**
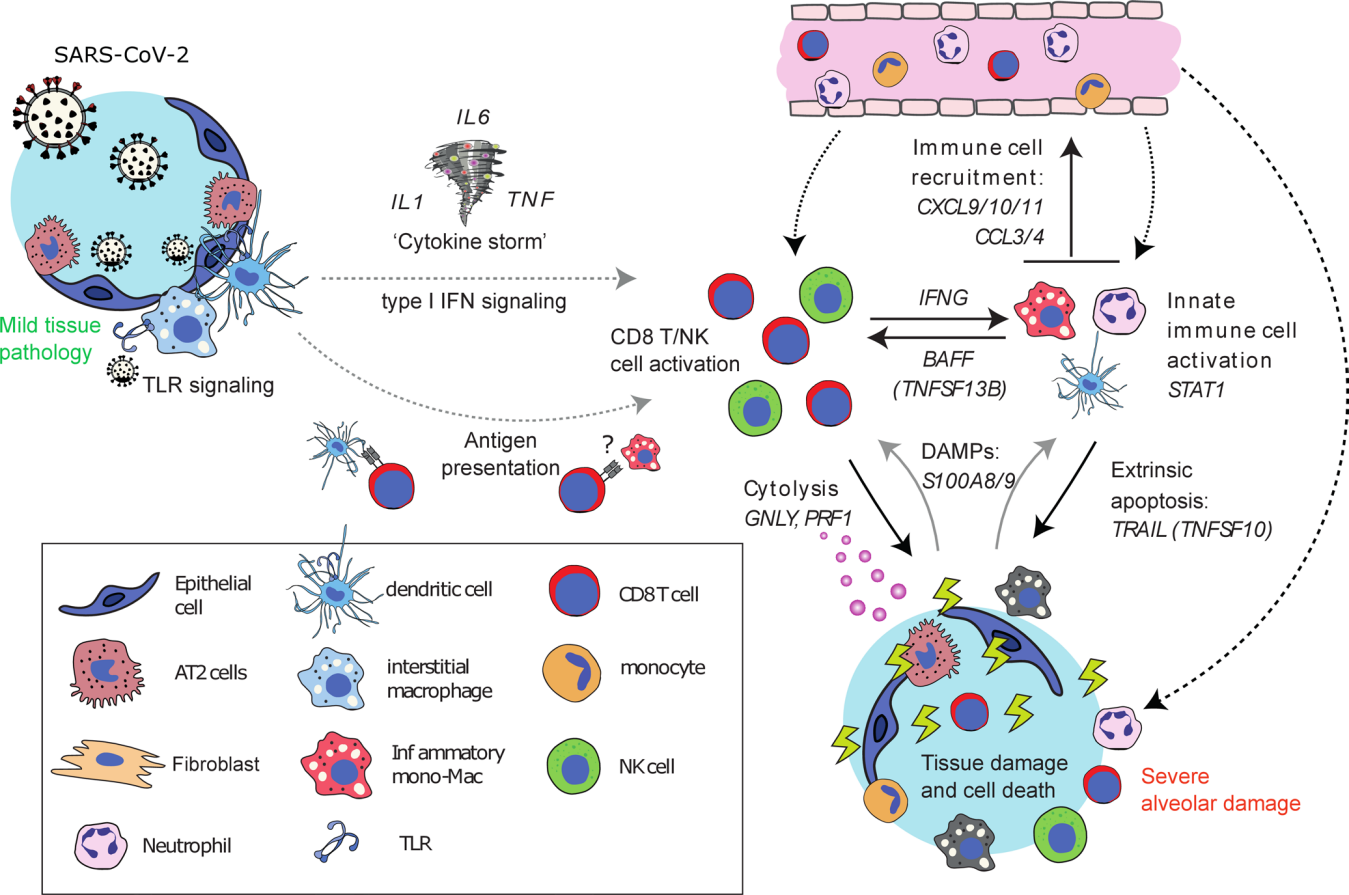
Proposed cellular model of severe tissue damage in COVID-19.

To further elucidate common features of severe COVID-19, we performed within-patient cellular phenotype network analysis and explored the reproducibility of our findings using data from published single-cell, single-nuclei, and spatial transcriptomic studies of COVID-19–infected tissues (6, 7, 18, 25). The results provide the basis of a model of severe lung tissue damage in COVID-19 in which IFN-γ production by CD8^+^ T and NK cells (a) activates macrophages and other innate immune cells and (b) induces expression of CXCL9/10/11 (15, 33), promoting further recruitment of CXCR3^+^ immune cells (including NK and cytotoxic T cells) into lymphoid-rich areas (Figure 6). The presence of cytotoxic lymphocytes and elevated expression of *TRAIL* (*TNFSF10*) suggests that severe lung damage in COVID-19 may involve cytolysis and extrinsically regulated apoptosis. In keeping with this model, myeloid cell dysregulation is a hallmark of severe or progressive COVID-19 infection (34–37), and there is strong evidence for increased numbers of macrophages in COVID-19 lung tissue (6, 7, 30). An increased ratio of CD14^+^HLA-DR^lo^ inflammatory monocytes to tissue-resident alveolar macrophages has also been noted (18), and macrophage hyperactivation by persistent IFN-γ production previously has been suggested to be a possible mechanism in COVID-19 (38). Consistent with the proposed model, despite the well-characterized peripheral blood T and NK cell lymphopenia (26), numbers of CD8^+^ T cells in COVID-19–infected lung tissue are comparable to those found in healthy individuals and higher than those found in pneumonia (6). NK cells are less abundant than CD8^+^ T cells in the lung tissue of patients with COVID-19 but appear to show an increase in mild disease that is reduced to, or below, healthy levels in severe cases (6, 7, 18). Our data extend these observations by showing that, within the lung tissue, cytotoxic CD8^+^ T cells can localize to interstitial immune cell infiltrates with inflammatory phenotypes, likely monoMacs and neutrophils, within areas of severe COVID-19–associated damage. On the basis of our transcriptomic analysis, we consider that such regions are also likely to contain cytotoxic NK cells, but we did not find transcriptional or immunohistochemical evidence for CD4 T cell involvement.

Our model is likely to be incomplete because of important limitations of our work, including the paucity of granulocyte detection in transcriptomic analysis, the number of patients studied, the targeted panel used for the transcriptomic analysis, and the limited spatial resolution of the GeoMx DSP platform. There were notable, patient-specific gene expression profiles, suggesting different states or stages of lethal COVID-19 pathology that may be associated with patient histories, differences in treatment and duration of sickness that could produce tissue differences in lung and SARS-CoV-2 interactions, the composition and distribution of inflammation, and the state of regenerative responses. More large-scale studies of COVID-19–infected lung tissue, using higher-resolution proteomic and whole-transcriptome spatial platforms to complement the throughput and targeted sampling that the GeoMx DSP platform affords, will be essential for fully deciphering the fine cellular and molecular details of inflammation and severe tissue damage in this disease (39).

Data from imaging and single-nuclei RNA-Seq studies in COVID-19 support a reduction in the proportion and absolute counts of type I and type II pneumocytes, with an expansion of transdifferentiating pneumocytes associated with a damage-associated epithelial progenitor phenotype (30, 40). In our study, subtypes of pneumocytes beyond epithelial cells and type II pneumocytes were not observed, but these may become apparent in larger spatial studies assessing the whole transcriptome. Pneumocyte gene expression and corresponding epithelial cells and type 2 pneumocytes signals were stronger in milder areas than in severe areas, although this may reflect a proportional increase in infiltrating and proliferating leukocytes or the loss of lineage-specific gene expression in damaged epithelium.

A key question that arises from the proposed model is about the nature of the upstream mechanism(s) by which CD8^+^ T and NK cells are stimulated to release IFN-γ in areas of severe damage. In a similar circuit proposed by Grant et al. (25), based on the analysis of BALF, activation of SARS-CoV-2–reactive T cells in lung alveoli was proposed to be sustained by continued SARS-CoV-2 infection of recruited monoMacs. However, the fact that only 1 of the 3 patients studied here had convincing evidence of viral infection in the tissue suggests that viral antigens may not be the only trigger for cytotoxic lymphocyte activation in severe cases. In support of this hypothesis, a similar observation was made in a study of lung tissue from fatal COVID-19 cases where “virus-independent immunopathology, rather than direct viral cytotoxicity” (8) was proposed to be a primary pathogenic mechanism. Additionally, it has also been reported that the CD8^+^ T cell repertoire is more diverse in BALF of patients with severe COVID-19 than in that of patients with moderate cases (18). Together, these observations suggest that in severe COVID-19, activation of CD8^+^ T cells in lung tissue may be spatially uncoupled from, and inappropriately sustained after, clearance of viral infection.

Candidate mechanisms for this process include exposure to endogenous DAMPs and/or pro-inflammatory cytokines. Possible endogenous DAMPs include the release of S100A8/9 from macrophages (and/or neutrophils) killed by cytotoxic lymphocytes or TRAIL-mediated extrinsic apoptosis if not adequately cleared by phagocytosis (41). With respect to the possible involvement of inflammatory cytokines, we noted prominent expression of *BAFF* (*TNFSF13B*) in regions of severe DAD, where, on the basis of inspection of single-cell data (18), the most likely source was the myeloid cells. BAFF can promote CD4^+^ T cell IFN-γ production and CD8^+^ T cell cytotoxicity in chronic obstructive pulmonary disease (42), supporting the concept that it may be part of a positive feedback loop that sustains nonspecific cytotoxic lymphocyte activation in COVID-19. It is also likely that elevated levels of IL-1, IL-6, and TNF may contribute to dysregulation of CD8 T cells, NK cells, and macrophages as part of the so-called cytokine storm (26, 43).

Currently deployed therapeutics such as dexamethasone and the anti–IL-6 therapy tocilizumab are effective but not curative in all patients (44). Although the cases we examined provided some evidence of a role for IL-6 in areas of milder damage, we did not find an obvious link between expression of this cytokine and severe tissue pathology. Our data suggest that therapeutic targeting of immune cell recruitment via the CXCL9– CXCL10–CXCL11/CXCR3 axis may be a valuable therapeutic strategy for resolution of inflammation in severe COVID-19, where it has become uncoupled from viral clearance. Systemic and lung tissue upregulation of these IFN-γ–induced cytokines in COVID-19 has been noted in many studies, and this axis has already been proposed by others as a therapeutic target for COVID-19 and other serious diseases, including cancer (6, 10, 15, 18, 26, 38, 45). Less attention has been paid to BAFF, which is markedly upregulated in plasma of patients with COVID-19, and for which monoclonal-blocking Abs have been developed (46, 47). Although more study of the role of this interesting cytokine in COVID-19 is required, our data suggest it may be a valid therapeutic target in severe cases. In 2 of the 3 patients studied, we also noted a robust upregulation of *IL32* in areas of severe damage, and the role of this cytokine, which has important functions in antiviral responses and can induce expression of pro-inflammatory cytokines, may warrant further study in COVID-19 (48).

Overall, the heterogeneity of the tissue pathology that we observed both within and between the patients studied underscores a need for personalized approaches in which the choice of therapy (or therapies) is guided by careful assessment of the stage of disease and viral clearance. For example, although innate immune activation via treatments such as intranasal IFNB1-α/β (49) are likely to be important for patients unable to clear the virus, our model predicts that they may exacerbate nonspecific lymphocyte and myeloid cell activation and tissue damage in virus-free lung tissue and in patients whose condition remains critical after successful viral clearance. As a more general strategy, our findings strongly support the use of combinatorial regimens that pair therapeutics directly targeting the virus, such as molnupiravir, remdesivir, ritonavir, and recombinant soluble ACE2 (50, 51), with those that can temper the dysregulated host immune responses that drive tissue damage.

## Methods

### Design.

To delineate the tissue-specific immune pathology in severe COVID-19, we assessed the transcriptomic profile across a spectrum of DAD in well-preserved tissue samples obtained at the point of death from 3 patients with COVID-19. Medical records of patients were retrospectively reviewed (52) and 3 patients with COVID-19 were selected for in-depth analysis on the basis of their clinical manifestation of acute respiratory distress syndrome (Supplemental Table 1), typical COVID-19 histology (Supplemental Table 2) with a 4–5 score on the Brescia–COVID Respiratory Severity Scale, and a lung-restricted presence of SARS-CoV-2 (i.e., absent in heart, liver, and kidney biopsy specimens). Patients with positive bacterial culture tests at or prior to death were excluded from this study. None of the patients were vaccinated against SARS-CoV-2. At least 14 AOIs from each COVID-19 tissue sample were selected for analysis, spanning, on average, 0.2 mm^2^ (range, 0.05–0.33 mm^2^) with exclusion of empty space. Areas were selected to represent the spectrum of alveolar injury within each tissue covering regions of (a) mild to moderate injury with some conservation of alveolar architecture and (b) severe injury with a loss of alveolar structure and substantial inflammation. Selected AOIs occupied alveolar and interstitial spaces, except from A_16 and A_17, containing bronchiolar epithelium. The severity grade of each AOI was confirmed post hoc by 2 pathologists.

### Patients and tissue processing.

Postmortem lung tissues were obtained through open biopsy at the point of death and processed as described (52). In brief, tissues were immediately fixed in neutral buffered formalin for less than 24 hours and then paraffin embedded. Sections (5 μm each) were cut for H&E staining, ISH, and DSP analysis. Six pathologists reviewed the histology and agreed on the gross histological characteristics (Supplemental Table 2). RNA was extracted from 5 μm sections (*n* = 4–8) for quantification of SARS-CoV-2 N and envelope protein transcripts (Supplemental Table 2).

### ISH.

ISH was conducted using the RNAscope2.5 LS Reagent Red Kit, according to the manufacturer’s instructions, and the Leica BOND-RXm system. Deparaffinization and heat-induced epitope retrieval were performed with BOND Epitope Retrieval Solution 2 (ER2; pH 9.0) for 25 minutes at 95°C. Hybridization for SARS-CoV-2 RNA was carried out using the RNAscope 2.5 LS Probe V-nCoV2019-S and checked for quality against slides treated with the positive control probe Hs-UBC and negative control Probe DapB. 

### IHC for SARS-CoV-2 N protein.

Deparaffinization and heat-induced epitope retrieval were performed on the Leica BOND-RXm using BOND ER2 (pH 9.0) for 30 minutes at 95°C.  Staining was conducted with the Bond Polymer Refine Detection kit and a rabbit anti–SARS-CoV-2 N Ab (Sinobiological; clone 001; dilution: 1:5000), and counterstaining was with hematoxylin. Whole-slide image analysis and QuPath software were used to quantify virus (53). Lung tissue was distinguished from empty space by applying the Create thresholder function on hematoxylin-stained areas, and then virus-positive pixels were quantified using the detect positive staining function (downsample factor: 10; Gaussian σ: 5 μm; hematoxylin threshold: 0.1 OD units; DAB threshold: 0.3 OD units).

### NanoString GeoMx digital spatial profiling.

This technique was carried out according to the manufacturer’s recommendations for GeoMx-NGS RNA BOND RX slide preparation (manual no. MAN-10131-02). Deparaffinization, rehydration, heat-induced epitope retrieval (for 20 minutes at 100°C), and enzymatic digestion (1 μg/mL proteinase K for 15 minutes at 37°C) were carried on the Leica BOND-RX. Tissues were incubated with 10% neutral buffered formalin for 5 minutes and for 5 minutes with NBF Stop buffer. The tissue sections were hybridized with the oligonucleotide probe mix (Cancer Transcriptome Atlas and COVID-19 spike-in panel) overnight, then blocked and incubated with PanCK-532 (clone AE1+AE3; Novus), CD3-647 (clone UMAB54; Origene), CD68-594 (clone KP1; Santa Cruz Biotechnology), and DNA dye (Syto13-488 dye; Invitrogen) for 1 hour. Tissue sections were then loaded into the GeoMx platform and scanned for an immunofluorescent signal.

After selection of AOIs, UV light directed at each AOI released oligonucleotides that were collected and prepared for sequencing. Illumina i5 and i7 dual-indexing primers were added during PCR (4 μL of collected oligonucleotide per AOI) to uniquely index each AOI. AMPure XP beads (Beckman Coulter) were used for PCR purification. Library concentration as measured using a Qubit fluorometer (Thermo Fisher Scientific), and quality was assessed using a Bioanalyzer (Agilent). Sequencing was performed on an Illumina NextSeq 2000, and FASTQ files were processed by the NanoString DND pipeline, resulting in count data for each target probe in each AOI.

### Analysis of immunofluorescent images for cell counts.

The number of nuclei and the CD3^+^ and CD68^+^ cell counts were determined using CellProfiler software. A pipeline was designed to quantify circular objects within RBG files for each AOI (54). Global manual intensity thresholds were set for object identification of nuclei and CD3^+^ cells, and CD68^+^ cells were identified by adapted intensity thresholds. The efficacy of object identification for each AOI was visually confirmed.

### Quality control and preprocessing of GeoMx transcript expression data.

Quality control and initial data exploration were conducted using the GeoMx DSP Analysis Suite. Sequencing quality per AOI was examined and an under-sequenced area (B_04) with zero deduplicated reads was excluded. Expression of each transcript was measured by 5 or more probes; outlier probes (defined as when the probe geomean in all AOIs divided by the geomean of all probes for a given target was <0.1, or probe failure in the Grubbs’ outlier test in >20% of AOIs) were excluded, and the remaining probes were combined to generate a single (after biological probe quality control) expression value per gene target per AOI.

We evaluated the performance of 2 normalization strategies, upper quartile and quantile normalization, by investigating their ability to standardize the expression distributions of housekeeping genes, negative control probes, and the full expression distribution between the AOIs. The expression values for negative control probes were not reported, because global outliers were appended to the gene expression matrix. On the basis of this assessment (and the results of subsequent principal component analyses [PCAs]), we proceeded with the quantile normalized expression values. We investigated the influence of known technical and biological factors on the variance structure of the data set by performing PCA of the log2(n+1) transformed quantile-normalized expression values. This analysis revealed that the first component in the data was associated with the aligned read-depth statistic. We therefore corrected the quantile normalized expression values for this technical factor using the Limma removeBatchEffect function.

Finally, to distinguish gene expression from background noise, we modeled the expression distribution of the set of negative probes, defining the median of the negative probe expression values plus 2 times the median absolute deviations as a robust detection threshold. In total, we retained 1631 genes that passed this detection threshold in at least 2 AOIs. The normalized expression distributions and sample PCA plots obtained after normalization, aligned read-depth correction, and expression-level filtering are shown in Supplemental Figure 3.

### Differential gene expression and overrepresentation analysis.

Differential gene expression was calculated for each gene between areas of mild to moderate and severe alveolar damage, using linear mixed models for repeated measures (dream methodology; R libraries: variancePartition, edgeR, and BiocParellel) designed to account for severity and aligned reads in filtered quantile-normalized data with patient identity as the random variable. *P* values were adjusted using the BH method. Overrepresentation analysis for GO BP terms was performed on genes with greater than 1.5-fold change and a FDR greater than 0.05 (R library: clusterProfiler; ref. 55) with the following parameters: pvalueCutoff = 0.05 and qvalueCutoff = 0.10. Redundant and duplicated pathways were removed for Figure 2B; for a full list of pathways, see Supplemental Table 3.

### WGCNA.

WGCNA was applied to the log2(n+1) transformed, quantile-normalized, aligned read-corrected and -filtered expression values. The parameter values were set as follows: the minimum fraction of nonmissing samples for a gene to be considered good was 0.5; the minimum number of nonmissing samples for a gene to be considered good was 4; the minimum number of good genes was 4; the cut height for removing outlying samples was 100 (no samples removed); the minimum number of objects on a branch to be considered a cluster was 2; network type equaled signed hybrid; soft power equaled 4; adjacency correlation function was bicor; adjacency distance was calculated by euclidean distance matrix computation; topological overlap matrix type was signed; minimum module size (number of genes) was 10; and the dissimilarity threshold used for merging modules was 0.25. The analysis identified 17 distinct modules labeled with colors, plus a module of 3 unassigned genes (grey module). The WGCNA analysis was performed using pipeline_wgcna.py (https://github.com/sansomlab/cornet; commit ID 196637b04d11682bc06670595f0ec1298a9aa1f4).

The expression patterns of the modules were summarized by calculation of module eigengenes using the WGCNA package (which defines a module’s eigengene as the first principal component of the expression of the module’s gene members). AOIs were hierarchically clustered by expression of the module eigengenes (Pearson’s correlation distance; optimized leaf ordering) from which 5 distinct groups were observed and highlighted using the R cutree function. The overrepresentation of GO categories, KEGG pathways, and reactome pathways in module gene members was tested using 1-sided Fisher’s exact tests (https://github.com/sansomlab/gsfisher; commit ID 3ad1d79293c6891cb23575e0e080fb61c74310b1), using the union of gene members from all the modules as the background gene set. Representative gene sets and pathways that had significant (BH adjusted *P* < 0.05) overrepresentations are displayed in the figures. WGCNA modules were further characterized by assessing the correlation of the module eigengenes with (a) histological severity (Pearson’s correlation), (b) predicted cell abundances (as estimated using SpatialDecon; Spearman’s correlation), and (c) that of selected genes (Spearman’s correlation; R library Hmisc).

### Cell deconvolution of GeoMx transcript expression data.

Deconvolution of cell types from the gene expression data was performed using the SpatialDecon R library (14). For this analysis, the full matrix of quantile-normalized gene counts (without correction), mean negative probe counts, and the cell profile matrix Lung_plus_neut were applied as input. The cell profile matrix retrieved from the package was generated from the Human Cell Atlas lung small conditional RNA-Seq data set and appended with neutrophil profiles, as described by Desai et al. (5). For correlation analyses, the abundance of cell types is reported for cell types present with greater than 2 relative abundance in more than 2 AOIs. Deconvoluted cell-type output is shown in Supplemental Figures 5 and 6 and Figures 3 and 4.

### Construction of AOI group-correlation networks.

We constructed correlation networks to investigate the relationship among the WGCNA modules, the estimated cell abundances (from SpatialDecon), and the expression levels of immune signaling genes for each of the 5 AOI spatial groups (R igraph library; layout = layout_with_dh). Nodes were scaled in size and/or color according to cell abundance, normalized gene expression, or WGCNA module eigengene expression. Drawn edges represent significant correlations (*P* < 0.05) and were weighted according to the value of the correlation coefficient (Spearman’s ρ). WGCNA modules were included in the network if they had a median module eigengene expression greater than zero within the relevant group of AOIs. Immune signaling genes from the KEGG cytokine-cytokine receptor interaction pathway (hsa04060; human) were included if they had a median expression above the expression detection threshold (as defined in the subsection *Quality control and preprocessing of GeoMx transcript expression data*) in a given AOI group. Cell-type abundance estimates were included if they had a median abundance of more than 2 of the given AOI group. For inclusion in the network, nodes (modules, cell types, and genes) had higher median values than the thresholds stated above and at least 1 positive correlation to another node type.

### Mass cytometry imaging.

FFPE lung tissue–section slides (5 μm thick) were stained with metal-conjugated Abs (anti–human CD68, CD4, CD8, and CD15; Fluidigm) after antigen retrieval. Intercalator-Ir (Fluidigm) was used to stain DNA. Slides were ablated on the Fluidigm Hyperion Imaging System using CyTOF7 software (Fluidigm) and visualized using an MCD Viewer (Fluidigm). Images were processed for publication using FIJI (56) to despeckle and sharpen the images.

### Data and material availability.

The NanoString GeoMx DSP raw sequencing data, the processed expression data, and the metadata are deposited at Gene Expression Omnibus series GSE186213. RBG composite images of the whole immunofluorescence-, IHC-, and H&E–stained tissue sections are available on Zenodo (10.5281/zenodo.7269953). Annotated scripts for the computational analysis are available from https://github.com/sansomlab/CrossJCIInsight2022; commit ID c604c5ce069f652324e5eeade26a3e23bbfe96d9.

### Statistics.

Statistics were carried out as indicated in the subsections *Differential gene expression and over-representation analysis*, *WGCNA*, *Cell deconvolution of GeoMx transcript expression data*, and *Construction of AOI group-correlation networks*.

### Study approval.

This study was approved by the ethics committee of the University of Navarra, Spain (approval no. 2020.192) and the Medical Sciences Interdivisional Research Ethics Committee of the University of Oxford (approval no. R76045/RE001). Tissues were stored at the John Radcliffe Hospital according to Human Tissue Authority regulations (License 12433).

## Author contributions

ARC, JH, PK, IM, and FI conceived the original experiments. CEDA, MFL, MVE, and IM obtained consent, clinical data, and the patient samples. ISDR and CEDA reported on tissue histology. ARC, LC, and FI designed and performed the experiments. SNS designed and supervised the computational analysis. SNS and ARC performed the computational analysis. ARC, FI, and SNS interpreted the results, generated the figures, acquired funding, oversaw the project, and wrote the manuscript with input from all of the authors. PW, REE, LD, GO, and LPH acquired funding for and designed and performed imaging mass spectrometry.

## Supplementary Material

Supplemental data

Supplemental tables 1-2

Supplemental table 3

Supplemental table 4

Supplemental table 5

Supplemental table 6

## Version 1 12/06/2022

In-Press Preview

## Version 2 01/24/2023

Electronic publication
